# An analysis of different modalities of bone mineral densitometry evaluation in cage subsidence in anterior cervical discectomy and fusion

**DOI:** 10.3389/fsurg.2024.1472080

**Published:** 2024-12-10

**Authors:** Qingsong Yu, Jiabao Chen, Haidong Wang, Lei Ma

**Affiliations:** Department of Spinal Surgery, The Third Hospital of Hebei Medical University, Shijiazhuang, China

**Keywords:** anterior cervical decompression and fusion (ACDF), cage subsidence, bone miner density, VBQ score, Dual energy Xray absorptiometry (DEXA)

## Abstract

**Objective:**

To compare the effectiveness of different measurement methods on bone miner density (BMD), including cervical HU of CT, MRI-based cervical vertebral bone quality (C-VBQ), and *T* value of DEXA, for predicting cage subsidence after single-level ACDF.

**Methods:**

This is a retrospective study, and patients who underwent single-level ACDF from June 2019 to June 2022 were recruited. We collected preoperative total segmental vertebral height (pre-TSVH), cage subsidence height, cervical angle (CA), T1-slope, straight or reverse cervical curvature, mean HU value of C3–7 (C-HU), mean HU of segment (seg-HU), C-VBQ, segmental C-VBQ (seg-VBQ), and total lumbar *T* value (*T* value). The variables between the two groups were compared by Student's *t* test or chi-square test. Logistic regression was used to determine the independent risk factors for subsidence. The ROC curve was used to analyze the predictive efficiency of C-HU, seg-HU, C-VBQ, seg-VBQ and *T* value for cage subsidence. Finally, the correlations of C-HU, seg-HU, C-VBQ, seg-VBQ, *T* value and subsidence height were analyzed.

**Results:**

A total of 320 patients were included in this study, and 97 patients (30.31%) had cage subsidence at the last follow-up. The subsidence height was 4.25 ± 0.937 mm in the subsidence group and 1.40 ± 0.726 mm in the nonsubsidence group. There were statistically significant differences between the two groups in bone mineral density-related indexes, including C-HU, seg-HU, C-VBQ, seg-VBQ, and *T* value (*p* < 0.05). Logistic regression analysis showed that C-HU was an independent risk factor for vertebral subsidence after single-level ACDF. ROC curve analysis showed that C-HU had the largest AUC of 0.897 (0.862, 0.933) in predicting vertebral subsidence. Correlation analysis showed that C-HU had a high correlation with the *T* value (*r* = 0.662, *p* < 0.001), while C-VBQ had a low correlation with the *T* value (*r* = −0.173, *p* = 0.002), and C-VBQ had a low correlation with subsidence height (*r* = 0.135, *p* = 0.016).

**Conclusion:**

Our study showed that compared with the C-VBQ and *T* value, C-HU is more effective for predicting cage subsidence after ACDF. Using the segmental index of C-VBQ or HU could not improve predictive effectiveness. C-VBQ may be insufficient in predicting cage subsidence and estimating BMD.

## Introduction

Anterior cervical discectomy and fusion (ACDF) surgery is a mainstream method for treating patients with degenerative cervical spinal diseases (1, 2). However, cage subsidence is the main factor affecting the long-term efficacy of surgery (3). It is associated with the recurrence of symptoms after an excellent clinical outcome and will lead to revision surgery (4). Although several factors have been reported to contribute to cage subsidence, including vertebral bone quality, age, cervical angle, cage material, bone graft material and quantity (5–7). Scholars agree that vertebral bone quality is the main factor affecting cage subsidence (6–8). However, the results of different measurement methods can show large differences. The measurement of bone miner density (BMD) may play a key role in predicting cage subsidence before surgery.

Dual energy x-ray absorptiometry (DEXA) and quantitative computed tomography (qCT) were the most common methods for assessing BMD (9, 10). qCT is an exact way to measure cervical vertebral BMD. However, it is not usually used in clinical examinations due to its high and extra cost and rare installation in hospitals. DEXA is usually used to test lumbar spine BMD, the indexes of the cervical spine are not clear, and the BMD relationship of the cervical and lumbar spine is uncertain (11).

Exploring an easy and efficient method based on routine radiological examinations as a supplement to qCT and DEXA for measuring cervical BMD and predicting the incidence of cage subsidence is of great significance for spine surgeons. Some studies revealed that lower vertebral Hounsfield units (HU) were correlated with deeper cage subsidence (6, 7). Based on MRI, a novel method of cervical vertebral bone quality (C-VBQ) was reported to be associated with cage subsidence (12). Nevertheless, the correlation between C-VBQ and BMD remains controversial. Lisa Oezel claimed that cervical VBQ scores may be insufficient in estimating BMD (13). Therefore, it is not clear which inspection method is better for finding the risk factors for cage subsidence. This study aimed to evaluate the correlation between cage subsidence and HU, C-VBQ, and DEXA results.

## Methods

### Patient population

This study was approved by the Ethical Committee of our hospital, and each patient signed an informed consent form. We retrospectively collected patient data in the Department of Spine Surgery of our hospital from June 2019 to June 2022. All patients underwent ACDF surgery, and operations were performed by chief physicians who had worked in spine surgery for more than 15 years. The inclusion criteria were as follows: 1. Diagnosis of degenerative cervical spinal diseases; 2. x-ray, DEXA, cervical CT, and cervical MRI were performed before surgery within 2 weeks; 3. Single-segment ACDF; exclusion criteria: 1. The internal fixation site was sheltered on x-ray; 2. The patients were not followed up on the 1st day, 3rd month, or 12th month after surgery. 3. Patients with spinal trauma, infection, tumor, or metabolic bone disease; 4. Patients with long-term use of hormones, including rheumatoid arthritis and autoimmune diseases. Electronic medical records were retrospectively queried to collect demographic data, including age, sex, body mass index (BMI), history of hypertension and diabetes, follow-up time, surgical segment, type of cervical spondylosis, and cage material.

### Radiographical assessment

All radiographical assessments were performed by two doctors who had been working in spine surgery for more than 5 years. They only evaluated imaging parameters and had no knowledge of the clinical information of the patient, and the two doctors were independent of each other and had no communication with each other.

Within 2 weeks before surgery, all patients completed radiographical examinations, including x-ray of the posteroanterior and lateral vertebrae, cervical CT and MRI, and the DEXA test of the lumbar spine. On the 1st day and at the 3rd and 12th months after surgery, all patients received posteroanterior and lateral x-rays. All data assessments were completed in the picture archiving and communication system (PACS) system.

### X-ray assessment and cage subsidence

**Figure 1:**
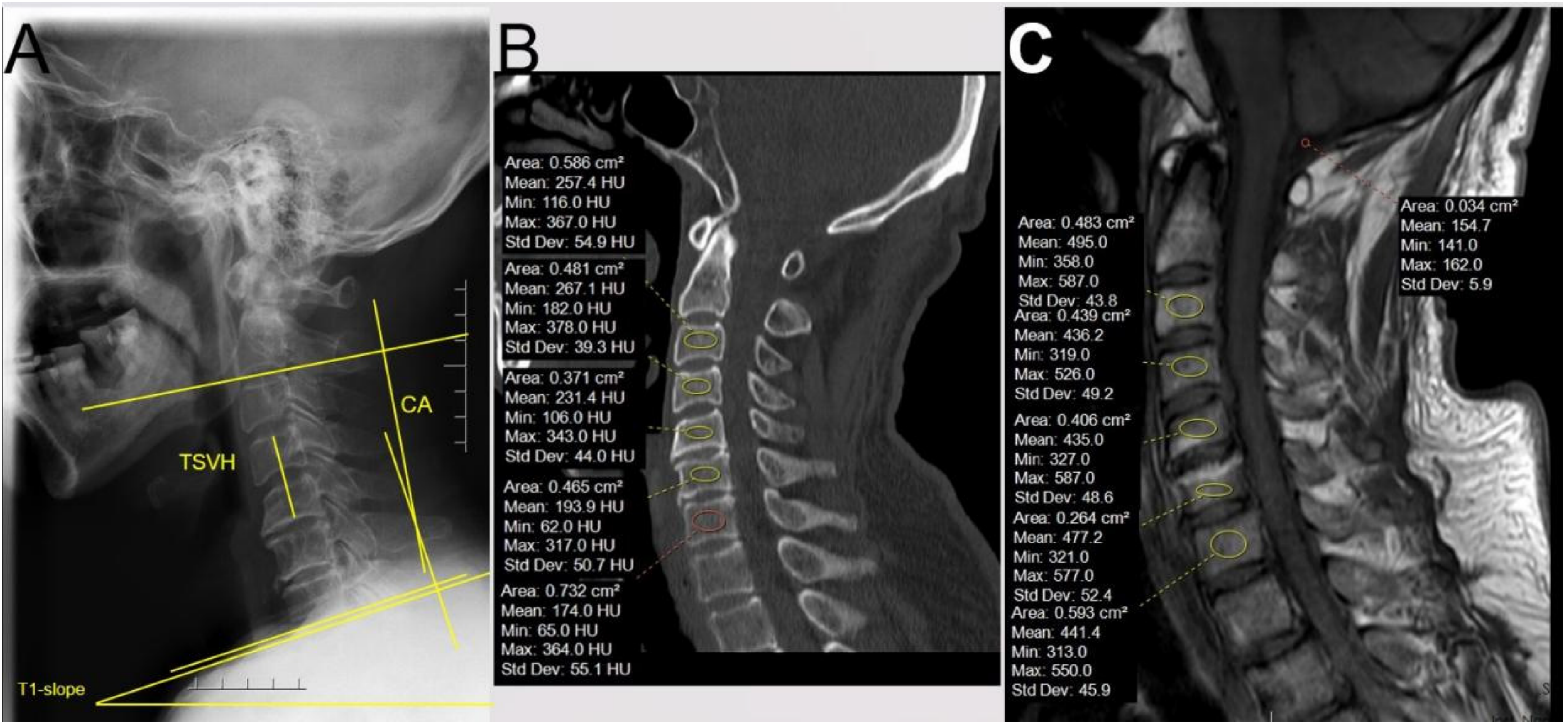
Preoperative measurement methods. **(A)** Different radiological parameters of x-ray. CA: the angle between the inferior endplate of C2 and the inferior endplate of C7. T1-slope: the angle between the superior endplate of T1 and the horizon. TSVH: the distance between the superior endplate of the upper vertebra and the inferior endplate of the lower vertebra of the surgical segment. **(B)** Measurement of Hounsfield units: the ROI was placed on cancellous, away from the endplate with more than 2 thicknesses of the endplate. **(C)** Measurement of C-VBQ: the ROI of the vertebra was the same as CT, and another ROI of cerebrospinal fluid was placed on the cisterna magna.

**Figure 2:**
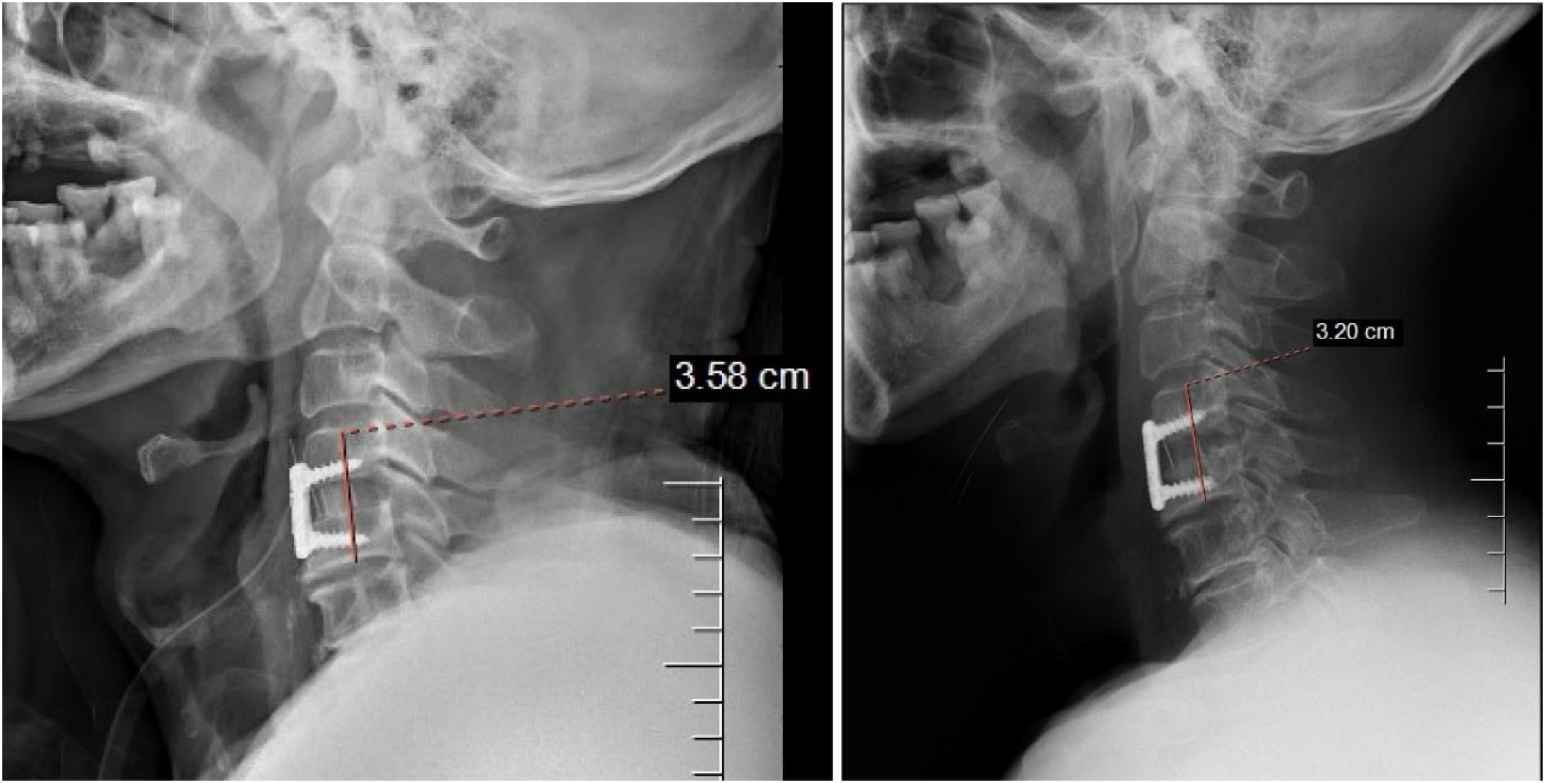
The change in TSVH postoperation. The figure on the left is the 1st day after surgery, and the right is the 12th month after surgery.

Radiographical parameters on plain radiographs before surgery included the C2–7 Cobb angle (CA), T1 slope, total segment vertebral height (TSVH) of the surgical segment, and straight or reverse cervical curvature (Figure 1). At the 1st day, 3rd and 12th month after surgery, we measured the TSVH of surgery. A TSVH reduction of ≥3 mm between the 1st day and the day after surgery or the last follow-up was defined as cage subsidence (14) (Figure 2).

### HU value calculation

All CT scans used a helical 64-channel CT scanner (SOMATOM Definition AS plus 128, Germany). Acquisitions were performed in helical mode with a tube voltage of 120 kVp, tube current of 50–220 mA, and slice thickness of 1.25 mm. The largest oval area of cancellous bone in the sagittal plane of the vertebral body was taken as the region of interest (ROI). We measured the HU of each ROI from C3–7 and calculated the average HU of the surgical segment (seg-HU) and the mean HU value of C3–7 (C-HU).

### C-VBQ calculation

Only T1-weighted images were studied. The cervical vertebral ROI was placed 3 mm from the perimeter of the vertebral body from C3–7. The CSF ROI was placed in an area within the cisterna magna. This measurement method was described by Lisa Oezel (13). Only 1.5 T MRIs were utilized for cervical VBQ calculations. We excluded C2 and T1 because these two segments were not included in the surgical segment. C-VBQ is equal to the mean value of the SI of C3–7 divided by the SI of CSF. The C-VBQ of the surgery segment (seg-VBQ) was equal to the mean value of the surgery segment divided by the SI of the CSF.

### *T* value of lumbar

Dual-energy x-ray absorptiometry (DEXA) scans were performed in lumbar vertebrae from 1–4 with a DEXA system (Hologic Discovery, Hologic, MA, USA). The *T* value of the total lumbar spine was recorded for this study.

### Statistical analysis

Statistical analysis of all data was performed using SPSS (version 23.0; SPSS Inc., Chicago, IL, USA). Statistical significance was set at *p* < 0.05. The ICCs (both inter- and intraobserver) of all variables were >0.75 and considered valid. The mean values of continuous variables were expressed as the means ± standard deviations (X ± SD) and compared using Student's *t* test between the subsidence and no-subsidence groups. The chi-square test with Fisher's exact test was used for categorical variables. Variables with *p* < 0.2 were included in a multiple logistic regression analysis to determine the predictive factors for subsidence. The sensitivity and specificity of the different BMD measurement methods for cage subsidence were compared using receiver operating characteristic (ROC) curve analysis. The scatter plot shows the relationship between the height of subsidence and the HU mean. The ICCs of different BMD measurement methods are shown in a scatter plot panel.

## Results

### Patient demographics

**Figure 3:**
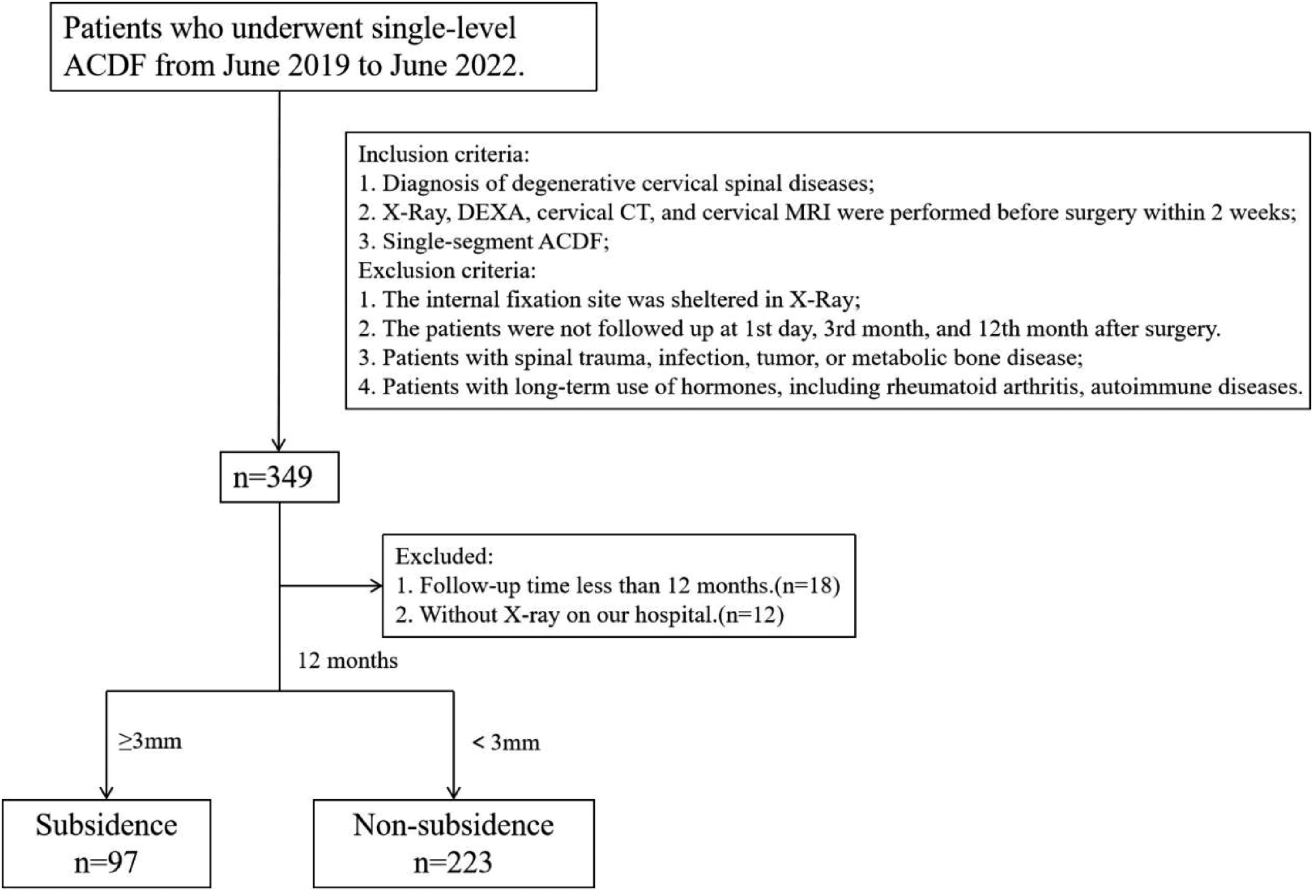
A flowchart of patients included in this study.

A total of 349 patients who underwent single-level ACDF surgery in our institution were retrospectively reviewed. Among them, 18 patients were lost to follow-up before completing 12 months postoperatively, and 11 patients were excluded because they did not complete the postoperative x-ray in our institution (Figure 3). The clinical information and imaging data of the remaining 320 patients were included in this investigation. They were divided into a subsidence group and a nonsubsidence group based on the presence or absence of cage subsidence at the last follow-up.

A total of 97 (30.31%) patients had cage subsidence. There were no significant differences in age, sex, BMI, hypertension, diabetes, follow-up time, surgical level, type of cervical spondylosis or fusion material between the two groups. The average degree of height loss in the subsidence group was 4.25 ± 0.937 mm, and that in the nonsubsidence group was 1.40 ± 0.726 mm (Table 1).

**Table 1:** Patients demographics.

	Subsidence	Nonsubsidence	*p* value
No. of patients	97	223	
Age(years)	62.10 ± 6.272	60.86 ± 7.318	0.124
Gender			0.344
Male	47	101	
Female	50	122	
BMI(kg/m^2^)	24.74 ± 3.453	24.77 ± 3.989	0.954
Hypertension			0.135
Yes	15	48	
No	82	175	
Diabetes			
Yes	13	31	0.524
No	84	191	
Fellow-up (month)	12.6 ± 0.66	12.6 ± 0.67	0.807
Surgery segment			0.496
C 3/4	7	28	
C 4/5	38	90	
C 5/6	44	88	
C 6/7	8	17	
Type of cervical spondylosis			0.797
Myelopathy	49	117	
Radiculopathy	14	36	
Mixed	34	70	
Cage material			0.137
Titanium	19	58	
PEEK	78	165	
Subsidence (mm)	4.25 ± 0.937	1.40 ± 0.726	<0.001

BMI, body mass index; mm, millimeter; PEEK, polyether ether ketone; pre TSVH, preoperative total segmental vertebral height.

### Risk factor analysis of cage subsidence

There was no significant difference in preoperative cervical curvature indexes (CA, T1-Slope and straight or reverse cervical curvature) between the two groups. However, the radiographic parameters related to BMD (C-HU, seg-HU, C-VBQ, seg-VBQ, and *T* value) showed significant differences between the two groups (Table 2).

**Table 2:** Compared the difference of radiographic parameters between the subsidence and nonsubsidence group.

	Subsidence	Nonsubsidence	*p* value
CA	13.7 ± 77.17	15.0 ± 8.23	0.142
T1-slope	21.5 ± 6.72	22.3 ± 8.45	0.374
Straight or reverse Cervical curvature	38	77	0.251
Pre TSVH (mm)	32.41 ± 2.550	32.51 ± 2.738	0.768
C-VBQ	2.98 ± 0.696	2.73 ± 0.569	0.02
seg-VBQ	2.99 ± 0.721	2.72 ± 0.598	0.01
C-HU	232.49 ± 35.052	319.19 ± 65.629	<0.001
seg-HU	240.56 ± 37.203	326.78 ± 73.771	<0.001
*T* value	−1.64 ± 0.825	−0.83 ± 0.838	<0.001

The variables with *p* < 0.2 (age, hypertension, cage material, CA, C-HU, seg-HU, C-VBQ, seg-VBQ, and *T* value) were included in the logistic regression analysis, and the results showed that C-HU was a single risk factor for cage subsidence (OR: 0.959, 95% CI: 0.949–0.969, *P* < 0.001) (Table 3).

**Table 3:** Risk factors for the cage subsidence in multiple regression analysis.

Variables	Univariate Analysis			Multivariate Analysis		
	B	*p*	OR(95% CI)	B	*p*	OR(95% CI)
Age	0.009	0.713	1.009 (0.961,1.060)			
Hypertension	−0.643	0.157	0.525 (0.215,1.281)			
Cage material	0.235	0.564	0.795 (0.359,1.760)			
CA	−0.002	0.919	0.998 (0.955,1.042)			
seg-VBQ	1.353	0.253	3.867 (0.38,39.363)			
C-VBQ	−0.050	0.265	0.251 (0.022,2.873)			
Seg-HU	0.007	0.369	1.007 (0.991,1.023)			
C-HU	−1.387	<0.001	0.951 (0.932,0.970)	−0.042	<0.001	0.959 (0.949, 0.969)
*T* value	0.105	0.671	1.110 (0.685,1.799)			

**Figure 4:**
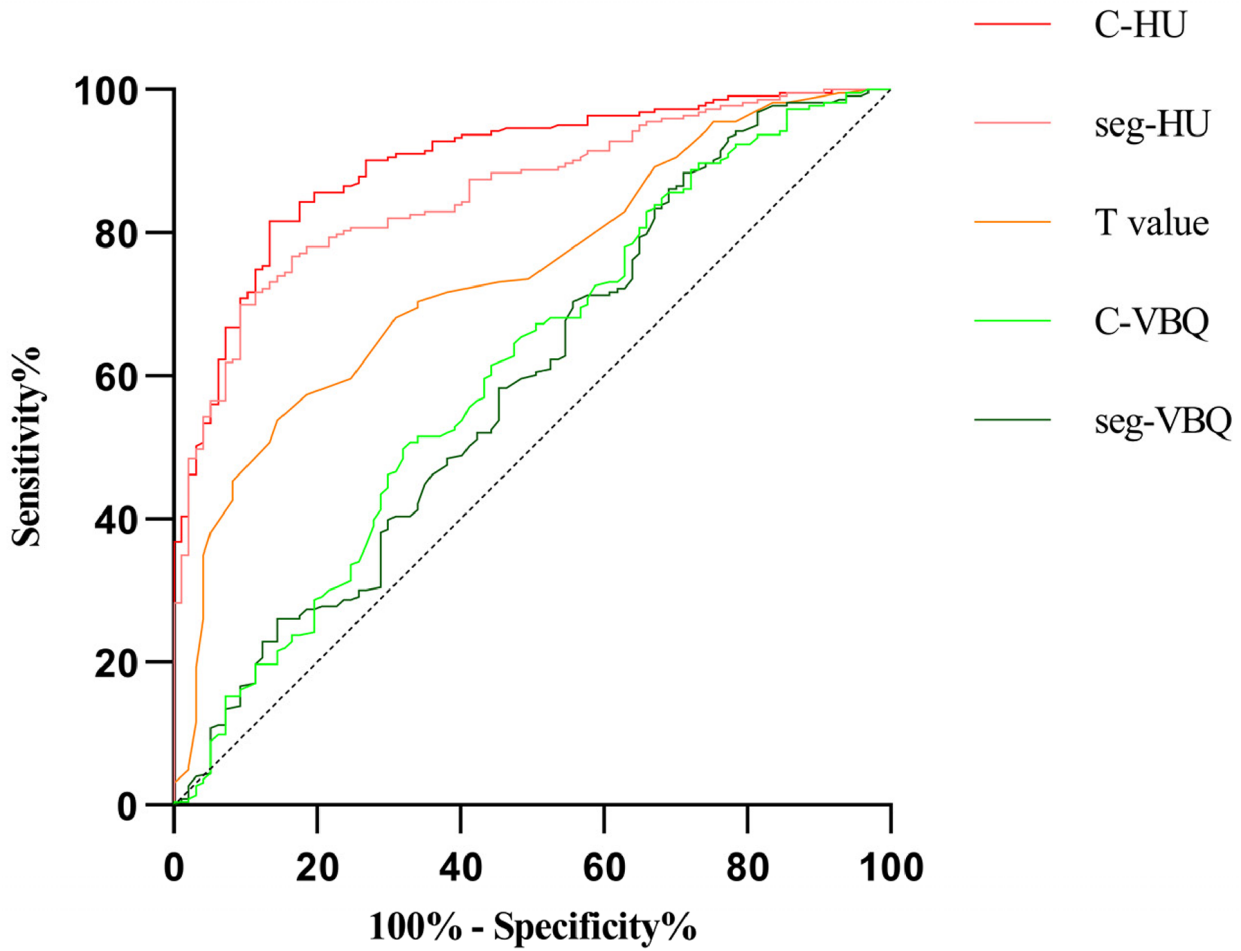
The ROC curves demonstrated the areas under the curve (AUCs) for different parameters of the BMD of the subsidence length.

The results of the ROC curve analysis showed that when C-HU was used to establish a prediction model, its sensitivity and specificity were higher than those of the *T* value and C-VBQ. The AUC of C-HU was 0.897. The AUC of the *T* value was 0.741, and that of the C-VBQ was 0.592, which was the lowest (Figure 4 and Table 4).

**Table 4:** Results of ROC curve analysis.

	Cut off	Sensitivity%	Specificity%	AUC(95% CI)	*p* value
C-HU	267.5	86.6	86.6	0.897 (0.862,0.933)	<0.001
seg-HU	283.0	69.96	90.72	0.857 (0.816,0.897)	<0.001
*T* value	−1.450	71.75	61.86	0.741 (0.685,0.798)	<0.001
C-VBQ	2.985	67.71	45.36	0.592 (0.522,0.622)	0.009
seg-VBQ	2.915	64.57	52.58	0.607 (0.538,0.676)	0.023

**Figure 5:**
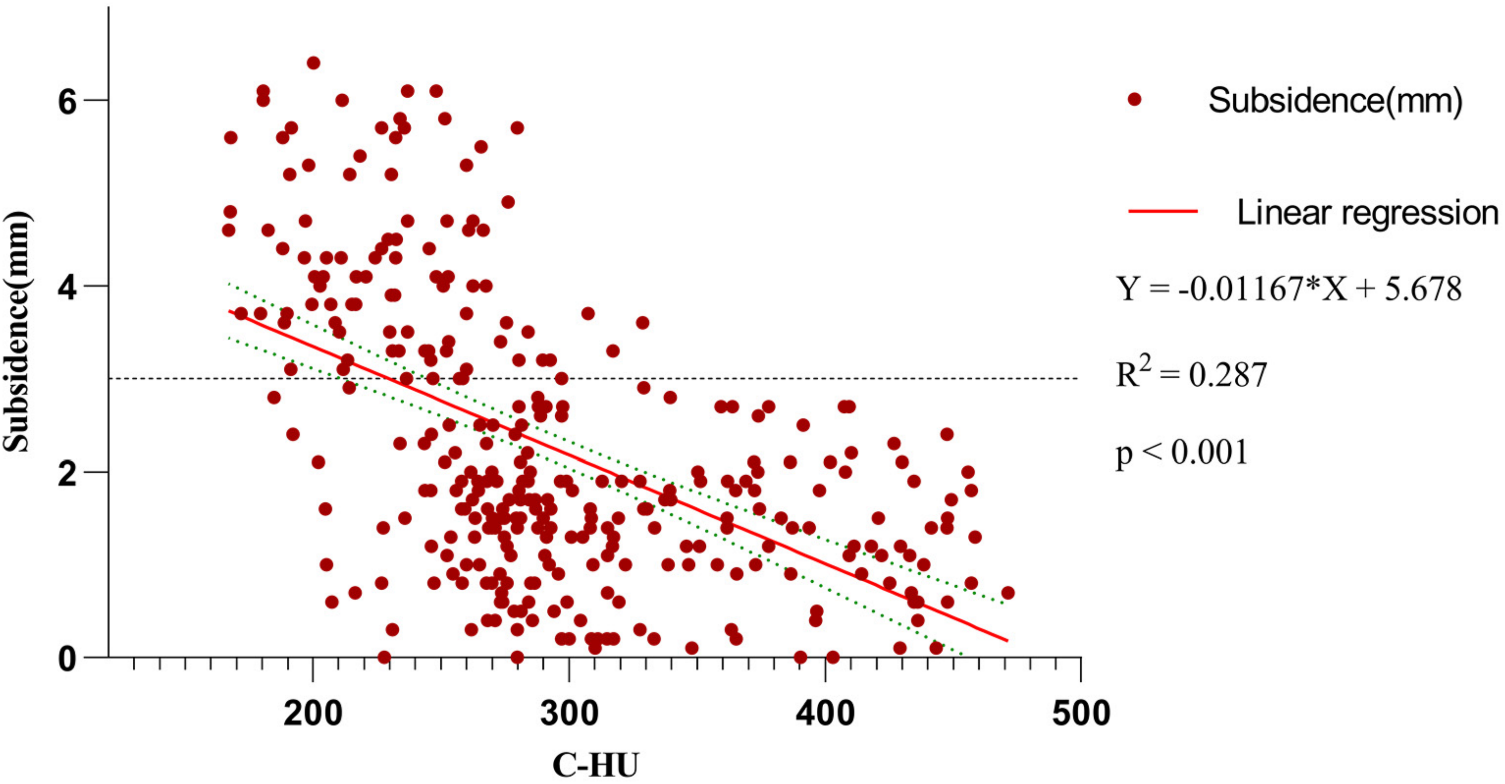
The graph demonstrates the relationship between C-HU and subsidence of TSVH.

Scatter plot between subsidence height and C-HU showed that subsidence of TSVH was negatively correlated with C-HU (*R*^2^ ^=^ ^0.287^, *p* < 0.001) (Figure 5).

### Correlation analysis of BMD measurement method

**Figure 6:**
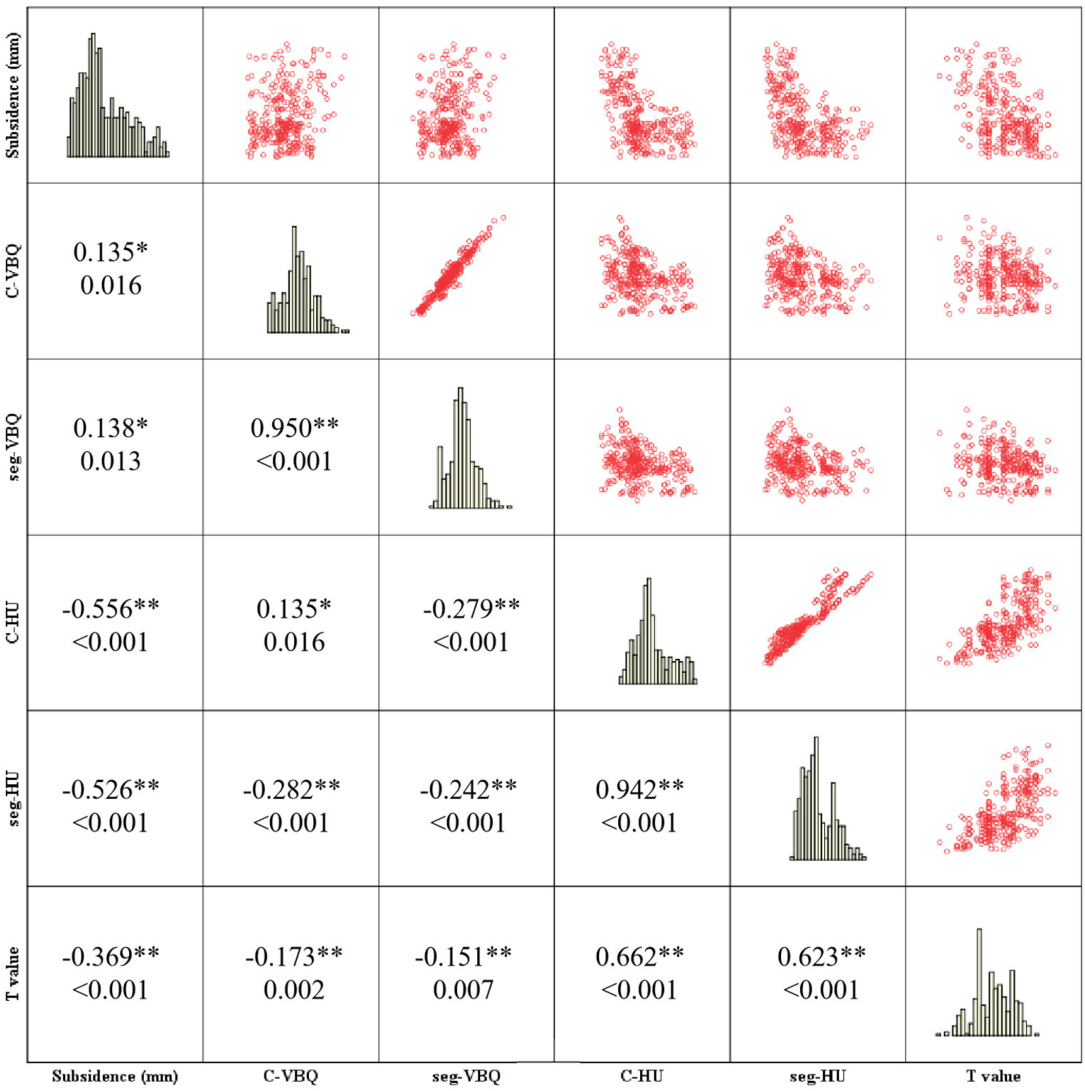
Correlation and scatter diagram matrix analysis showed the relationship between subsidence of TSVH and BMD parameters. The first number in the small square represents the Spearman's correlation coefficient, and the other number represents the *p* value.

The correlation analysis and scatter plot panel (Figure 6) showed the distribution relationship and correlation among C-VBQ, C-HU, *T* value and subsidence height. C-HU is positively correlated with the *T* value, which can better reflect the BMD of the cervical spine. However, the correlation between C-VBQ and *T* value and C-HU is weak, which may be insufficient to evaluate the cervical spine BMD of patients. The correlation between subsidence height and C-VBQ was also weak. This result suggested that the value of C-VBQ as a predictor of cage subsidence was inaccurate and much lower than that of C-HU. Before surgery, the C-HU value should be used as a parameter to evaluate the BMD of the cervical spine and as a risk predictor of cage subsidence.

## Discussion

ACDF can achieve nerve decompression, maintaining the intervertebral space and the stability of the cervical spine. However, cage subsidence after surgery is a common phenomenon that may lead to the recurrence of symptoms after surgery and even to new neurological symptoms (15, 16). The highest incidence of cage subsidence is reported to be 83% (17). At present, there is no consensus for cage subsidence standards. A subsidence height ≥3 mm is usually regarded as the critical standard of subsidence, which has high accuracy in predicting patients with subsidence complications (14, 18).

In recent years, many studies have focused on the risk factors of cage subsidence, trying to find an appropriate assessment method to reduce the risk of vertebral body subsidence. Previous studies have suggested that cage settlement and bone quality, cage material, multi-level ACDF, anterior cervical locking plate, preoperative cervical curvature and other factors (5, 19). Most studies will collectively consider bone mineral density as a high risk factor for cage subsidence. Patients with low bone mineral density, poor vertebral strength, can not provide effective support for the cage. This leads to the occurrence of vertebral subsidence.

The appearance of low BMD is the most significant risk factor for cage subsidence and looseness and displacement of fixations. In clinical work, when the patient has osteoporosis, anti-osteoporosis treatment should be chosen first, and surgical treatment should often be performed after the improvement of osteoporosis. As shown in Table 2, the C-HU, C-VBQ, and *T* values were different between the subsidence and nonsubsidence groups.

According to the World Health Organization (WHO) recommendation, the gold standard diagnostic criterion of osteoporosis was a *T* value ≤ −2.5 standard deviations in the DEXA test (20, 21). It represents the bone miner content of the whole body and is useful for orthopedic surgeons to estimate the BMD of patients and decide the type of operation. However, it cannot exactly reflect the BMD of cervical vertebrae due to the lack of cervical BMD data. A few hospitals use qCT as a BMD measuring machine, which can obtain the exact BMD of cervical vertebrae (22–24). However, most hospitals cannot equip qCT for exorbitant price and extra radiation.

'The x-ray test and CT and MRI scans should be completed before ACDF as common radiographical tests, and those devices are equipped in most hospitals. Whether these examinations can be used to evaluate the BMD of patients and predict the probability of cage subsidence has been a research hotspot. According to previous reports, x-ray could be used to differentiate osteoporosis and normal BMD, but it was replaced by DEXA for lower accuracy (25). The HU value was related to BMD, which was observed in the lumbar spine, thoracic spine, cervical spine, and other bones of the body (26). In some recent studies, it was shown that HU was correlated with BMD (27, 28), and lower HU indicated a high risk of graft subsidence (7, 8, 29). As a novel predictor of BMD, VBQ was established by Ehresman et al., which can identify healthy and osteopenic/osteoporotic bone with an accuracy of 81% in the lumbar spine. Hu YH et al. claimed that VBQ was a good predictor of cage subsidence after lumbar fusion surgery (30). Lisa Oezel et al. first investigated the relationship between C-VBQ and qCT of every vertebra and total cervical vertebra. They found that C-VBQ is not sufficient to assess cervical BMDs, which may limit the clinical application of C-VBQ (13). Cathleen C Kuo et al. showed that C-VBQ is highly correlated with lumbar VBQ (31). Soliman MAR et al. first declared the relationship between C-VBQ and cage subsidence. They found that higher C-VBQ is significantly associated with cage subsidence. However, the sample size was limited to only 59 patients and did not provide clarification on which predictor, HU or C-VBQ, was more effective in predicting cage subsidence after ACDF (12). According to James T. Bernatz et al., when the threshold value of C-VBQ is 3.2, C-VBQ can predict subsidence very accurately, and the AUC value reached 0.99. However, the number of patients included in this report was small, with 44 patients, and only 10 patients had subsidence (32). In another study, the measurement method of C-VBQ was modified, and the VBQ value of the cervical endplate was considered to be a better predictor of the subsidence after cervical spine surgery. However, due to the small and concave shape of the cervical endplate on MRI, it seems difficult to select the accurate region of the endplate by this rectangular selection method, and there may be a large measurement error, which limits the promotion of this measurement method (33).

As we all know, no study has revealed a correlation between DEXA results and C-VBQ, and the threshold of those tests that can predict cage subsidence is still not clear. This study is the first to comprehensively compare the different radiographical parameters (including CT, MRI, and DEXA) indicating patients' BMD.

We found that patients with cage subsidence had lower preoperative parameters in C-HU or seg-HU and *T* value and higher parameters in C-VBQ or seg-VBQ than those in patients without cage subsidence. There were statistically significant differences (*p* < 0.05). The HU value, *T* value and C-VBQ have collinearity in predicting cage subsidence. Logistic regression analysis showed that only C-HU was an independent risk factor for cage subsidence. This means that the C-HU value can be used to establish a single predictive model for cage subsidence after ACDF. The ROC curve also showed that C-HU had the largest AUC area (0.897, CI: 0.862–0.933), and the predictive effect was the best. When C-HU was lower than 267.5, the risk of postoperative cage subsidence was the highest, and the probability of miscalculation was the lowest. The seg-HU was close to C-HU. However, the ROC curve showed that the predictive value of C-VBQ was very low (0.592, CI: 0.522–0.662). The scatter plot further revealed that there was a negative correlation between cage settlement height and C-HU.

Correlation analysis showed that C-HU and seg-HU were moderately positively correlated with the *T* value of the lumbar spine, while the correlation coefficients of C-VBQ and seg-VBQ with the *T* value were low, which could be regarded as no linear correlation between them. Although DEXA is the gold standard for BMD measurement, the bone distribution of the lumbar spine is significantly different from that of the cervical spine. This may explain why the test of lumbar *T* value has a poorer efficacy than the test of cervical mean HU in predicting vertebral subsidence. This also means that the BMD of cervical trabecular bone may be a decisive risk factor for vertebral subsidence after ACDF. Although there was a difference in C-VBQ and seg-VBQ between the subsidence and nonsubsidence groups, such an insignificant difference was not sufficient to make it a discriminator of cage subsidence. Therefore, the HU value of CT should be measured to evaluate the risk of cage subsidence during preoperative planning.

There are limitations in this study. First, this study is a single-center retrospective study, and multicenter and prospective studies will be performed in the future. Second, because qCT was not used as a common examination in our hospital, HU and C-VBQ values with BMD of the lumbar spine were chosen as the study parameters. Third, the cage size and intraoperative intervertebral traction distance were not included in the study. Last, it should be pointed out that we only analyzed the subsidence results of the standard single-segment ACDF surgery. All patients used titanium plates and internal fixation screws, and patients chose 3D-printed Titanium or PEEK materials cage according to their personal wishes and economic conditions. It would not be able to draw the same conclusions for other types of interbody material or anterior surgeries. Such as anterior cervical corpectomy and fusion, multi-level ACDF surgery, artificial disc replacement surgery and Zero-profile anterior cervical interbody fusion.

In conclusion, preoperative assessment of cervical spine BMD is an important means to predict vertebral subsidence before patients undergo ACDF surgery. The C-VBQ had very little predictive efficacy on cage subsidence. Cervical CT outperforms DEXA and MRI in predicting cage subsidence, and a low mean cervical HU value should be considered a predictor for cage subsidence after surgery.

## Data Availability

The raw data supporting the conclusions of this article will be made available by the authors, without undue reservation.
